# Video Q&A: Rapid urine LAM test for HIV-associated TB - potential to reduce deaths? An interview with Stephen Lawn

**DOI:** 10.1186/1741-7015-11-158

**Published:** 2013-07-04

**Authors:** Stephen D Lawn

**Affiliations:** 1 Department of Clinical Research, Faculty of Infectious and Tropical Diseases, London School of Hygiene and Tropical Medicine, Keppel Street, London, WC1E 7HT, UK; 2 Desmond Tutu HIV Centre, Institute of Infectious Disease and Molecular Medicine, University of Cape Town, Cape Town, South Africa

## Abstract

In this video Q&A, we talk to Dr Stephen Lawn about the point-of-care LAM test for HIV-associated TB, which has the potential to save lives by improving rapid diagnosis and treatment.

## An interview with Stephen Lawn

## Introduction

Stephen Lawn trained in medicine at Nottingham University and specialised in Infectious Diseases in London in the UK. At various stages of his career as a clinician scientist, he has worked in Ghana in West Africa, the Centers for Diseases Control and Prevention in Atlanta, USA, and at the University of Cape Town in South Africa. He is a Reader and Consultant Physician in Infectious Diseases at the London School of Hygiene & Tropical Medicine and an Associate Professor of Infectious Diseases and HIV Medicine at the University of Cape Town. His major research interests are in HIV infection and tuberculosis (TB). In Cape Town, he has conducted studies evaluating novel assays and approaches to screening and diagnosis of HIV-associated TB.

Stephen Lawn has no conflicts of interest to declare in relation to any of the diagnostic tests referred to in this article.

## Transcript

### 1. What is the scale of the global HIV-associated tuberculosis (TB) epidemic?

The World Health Organization estimated that in 2011 there were 1.1 million new or recurrent cases of TB in people living with HIV worldwide, which is around 13% of total TB cases. This burden of HIV-associated TB is highly concentrated in the countries of sub-Saharan Africa, which account for 79% of all cases. Disease rates are highest in countries towards the south of the continent where HIV prevalence is greatest. Here between 50% and 80% of TB cases are HIV-co infected. One country alone, South Africa, accounts for almost 30% of all cases worldwide. Outside Africa, TB is also a common opportunistic infection in people living with HIV in south-east Asia and South America and among HIV-infected injection drug users in the countries of eastern Europe and central Asia.

HIV-associated TB causes approx 430,000 deaths per year, although these are classified as HIV deaths in the International Classification of Diseases. Approximately one third of people who die with TB are HIV-co infected and approximately 25% of global HIV/AIDS deaths have TB as the underlying cause. So, TB is the leading cause of death in people with HIV/AIDS worldwide.

### 2. Why is diagnosis of TB more challenging in people living with HIV infection, especially in resource-limited settings?

Diagnosis of TB in resource-limited settings relies heavily on sputum smear microscopy and chest radiology, both of which are impaired in people with HIV co infection. TB diagnosis by sputum smear microscopy depends on the release of TB bacilli (*Mycobacterium tuberculosis*) in sufficient numbers into the sputum such that the concentration of organisms exceeds 10,000 bacilli per ml of sputum, which is the limit of detection of the assay. However, HIV impairs antimycobacterial immune responses so that co-infected patients have reduced immunopathology in the lungs, which means that there is less inflammation and less lung tissue damage. As a result, lower concentrations of bacilli are liberated into sputum and so sputum smears are much more likely to be negative. This issue is further compounded by the fact that when patients have advanced disease and are very weak, it can be very difficult for them to expectorate good quality sputum samples.

In addition to sputum smear microscopy, chest radiology is less useful in those with HIV co infection. Again, because of reduced immunopathology, the chest radiographic appearances are often non-specific and lack the typical characteristics of pulmonary TB seen in HIV-negative patients. HIV co-infection also increases the frequency of extra-pulmonary disease which further compounds the challenge of diagnosis.

The huge challenge of diagnosis of HIV-associated TB is graphically illustrated by a number of post-mortem studies of patients with HIV/AIDS who have died in hospitals in sub-Saharan Africa. These studies have repeatedly shown that between 30% and 50% of patients had evidence of TB, much of which is disseminated and remained undiagnosed at the time of death.

### 3. What other laboratory tests may help improve diagnosis of HIV-associated TB?

Mycobacterial culture of samples, especially in liquid media, is the assay with the highest sensitivity for TB diagnosis. However, this is slow, often yielding results in weeks rather than days. It is technologically demanding and expensive and is only feasible in centralised laboratories. For these reasons it is not generally available in many resource-limited settings.

However, a real landmark development is the Xpert MTB/RIF assay which was endorsed by WHO in December 2010. This is a simplified fully automated real-time PCR assay system that is cartridge-based and requires very limited training for operation. It takes just two hours to generate a result and it has much higher sensitivity that sputum smear microscopy. A single test can detect all smear-positive cases and approximately 70%-75% of smear-negative cases. However, there are a number of draw-backs. The hardware is sophisticated and expensive. Each cartridge, even at heavily subsidized prices, costs $10. Moreover, its use will largely be confined to laboratory settings as it is difficult to implement at the point-of-care. When use is laboratory-based, results cannot be used to immediately inform treatment decisions.

What is really needed is a low-cost point-of-care assay that can be used to reliably diagnose TB and permit TB treatment to be started at the same clinic visit. The Determine TB-LAM assay is one such test that has recently become commercially available. Although this has not yet been endorsed and more evidence is needed from ongoing studies, this assay has real potential to play a useful role in diagnosis of HIV-associated TB.

### 4. What is the basis for TB diagnosis using the urine lipoarabinomannan (LAM) antigen detection?

Lipoarabinomannan (LAM) is a glycolipid component of the cell wall of *Mycobacterium tuberculosis*. It is produced in large quantities by the organism and this may relate to the fact that it is immunomodulatory, favouring survival of the organism in the human host. LAM can be detected in the urine of a proportion of patients with TB, even if the primary focus of the disease is, for example, pulmonary. LAM is thought to gain entry to the urine either via the bloodstream or via direct involvement of the renal tract by TB in those with disseminated disease. Regardless of the mechanism, detection of LAM in urine can be used as a means of TB diagnosis.

TB diagnosis via detection of LAM in urine can be done in the laboratory using a simple polyclonal antibody sandwich ELISA and the initial diagnostic accuracy studies were done using this format of the assay. The ELISA has now been developed into a simple lateral-flow ‘strip-test’ format – which is an immunochromatographic assay that looks like a urine pregnancy test.

The test requires a fresh urine sample and just 60 μL is applied to the sample pad at the bottom of the strip without any prior processing required. The strip is left for 25 minutes during which time the urine containing LAM travels up the test strip and immobilised capture and detection antibodies labelled with colloidal gold lead to the development of a purple band in the test window. If the band is of sufficient intensity when compared with a reference card, it is scored as a positive test for LAM.

### 5. In which patients is the urine LAM test useful?

A series of studies published since 2009 have shown that the utility of LAM detection for TB diagnosis is restricted to patients with HIV infection and advanced immunodeficiency. It is not useful in HIV-negative patients and it is not useful in HIV-positive patients with CD4 counts >200 cells/μL. So this test should not be applied to unselected patients.

However, among HIV-infected patients with CD4 counts <200 cells/μL the test is useful. The lower the CD4 cell counts and the sicker the patients (as defined by a range of characteristics), the greater the sensitivity for TB diagnosis. The likely reason for this is that such patients have a much higher risk of disseminated disease and therefore a greater likelihood of LAM being present in urine.

The sensitivity of the assay entirely depends on the particular characteristics of the patients tested. Among ambulatory out-patients with CD4 cell counts <50 cells/μL, the test detects approximately two-thirds of cases. Among in-patients who are of course sicker, this proportion may be higher. Conversely, among ambulatory patients with higher CD4 counts, the sensitivity will be lower.

### 6. What are the advantages and disadvantages of this test?

The major advantages of this test are obvious. The test is low-cost (currently marketed at $3.50 per test), rapid, and doesn’t require any equipment. Urine is easy to obtain even among sick patients in hospital who may find it difficult to produce sputum samples. Urine is safe and easy to handle and testing could readily be done by nursing staff following limited training. Most importantly, it can be used at the point-of-care, permitting diagnosis and start of treatment at a single clinic visit, potentially reducing treatment delays greatly.

The disadvantages of the test are that it should only be used among those who are confirmed to be HIV-infected and to have advanced immunodeficiency as defined by CD4 count or WHO clinical staging. The sensitivity of the assay is limited and so it can only be used as a test to diagnose TB but cannot be used as a test to rule-out TB. While several well-conducted studies of LAM ELISA and point-of-care assays have found very high assay specificity, some studies have found more limited specificity. Whether this is true or an artefact of study design remains to be fully clarified. Finally, the optimal cut-off for scoring the point-of-care strip tests as positive or negative has yet to be finalised. This needs to take into account both its ease of use by health-care workers as well as diagnostic accuracy.

So a range of further studies are needed to clarify the use of the Determine TB-LAM point-of-care assay. These must increase the evidence base on the diagnostic accuracy of this assay, operational research is needed on its use in the clinical rather than the laboratory environment and finally, large-scale studies are needed to assess the impact of implementation on clinical outcomes.

### 7. What role might this assay play in diagnosis of HIV-associated TB in resource-limited settings?

There are several characteristics of the Determine TB-LAM point-of-care assay that makes it potentially very useful. First, the really key observation is that although the sensitivity of the assay is somewhat limited, sensitivity is highest in the very sub-set of patients who have the worst prognostic characteristics and are at greatest risk of death. Secondly, the assay permits TB diagnosis during a single clinic visit and immediate initiation of treatment. So there is the real potential to accelerate diagnosis and treatment and thereby reduce mortality risk.

The assay is not a stand-alone assay but should be used in combination with other assays. Indeed, there is incremental sensitivity when used in combination with sputum smear microscopy or sputum Xpert testing. Thus, the LAM assay shortens the time to diagnosis in the sickest patients and increases overall sensitivity when used in combination with other assays. So this assay may play an important role in the diagnostic algorithm.

There are two key clinical populations in which this assay may come to play an important role. First, the assay may be used to screen for TB in HIV-infected out-patients with advanced immunodeficiency before they start antiretroviral therapy. Second, it can be used for screening and diagnosis of TB in HIV-infected in-patients. By shortening the time to diagnosis in these key patient groups, we would hope that their mortality risk would be reduced. So the real goal and potential of this assay is to reduce deaths from HIV-associated TB.

### 8. Where can I find out more?

See references [1-10].

## Note

This article is part of the article collections Medicine for Global Health (http://www.biomedcentral.com/bmcmed/series/MGH) and HIV thirty years on (http://www.biomedcentral.com/series/HIV_30)

## Pre-publication history

The pre-publication history for this paper can be accessed here:

http://www.biomedcentral.com/1741-7015/11/158/prepub

## Supplementary Material

An interview with Stephen Lawn Click here for file
